# Cross-cultural adaptation and validation of Dysphagia Handicap Index in Bangladesh

**DOI:** 10.1186/s41687-024-00803-y

**Published:** 2025-01-02

**Authors:** Mehrin Sultana, Md. Muid Hossain Reshad, Md. Shohidul Islam Mridha

**Affiliations:** 1 Centre for the Rehabilitation of the Paralysed (CRP), Dhaka, Bangladesh; 2 Department of SLT, Bangladesh Health Professions Institute (BHPI), CRP, Dhaka, Bangladesh

**Keywords:** Cross-cultural adaptation, Linguistic validation, Dysphasia Handicap Index (DHI), Dysphagia, Handicap, Patient-reported outcome, Quality of life (QoL)

## Abstract

The Dysphagia Handicap Index (DHI) is commonly utilized for evaluating how dysphagia impacts the quality of life (QoL) of patients across physical, functional, and emotional dimensions. The primary aim of the research was to linguistically validate and culturally adapt the DHI to the Bangla version. A cross-sectional study design was chosen, with Beaton’s protocol as the guiding framework for validating and adapting the DHI. It has followed a systematic process of forward translation, participation in expert discussions, and subsequent back translation to obtain a reviewed version. The Bangla version, DHI-Ban, was administered purposefully to 50 dysphagia patients in the Clinical Speech and Language Therapy (SLT) Department of the Centre for the Rehabilitation of the Paralyzed (CRP) and was also administered to 50 healthy individuals for comparison. Of the fifty, eighteen dysphagia subjects were assigned again after two weeks for the retest. The DHI-Ban demonstrated strong internal consistency (Cronbach’s *α* = 0.89) and good test-retest reproducibility (ICC = 0.86). The Spearman test confirmed significant construct validity (*p* < 0.01), and the Wilcoxon test identified significant differences (*p* < 0.001) between patients and healthy individuals. Feedback from participants was also taken into account for acceptance and clarity. In conclusion, the adapted DHI-Ban has emerged to be a reliable patient-reported tool for assessing dysphagia in Bangla-speaking individuals. Incorporating the Bangla language framework facilitates its comprehension and effectiveness, further solidifying its reliability.

## Introduction

Dysphagia, which results from abnormalities in the intricate neuromuscular swallowing process, dramatically lowers the quality of life (QoL) [1–4] and frequently points to underlying primary medical conditions, most commonly neurological or structural disorders [5]. Inadequate swallowing can result in malnourishment, dehydration, and an elevated risk of aspiration [2, 6]. It is linked to several health issues, including stroke, head and neck cancer, cerebral palsy, dementia, pneumonia, complications from surgery or radiation therapy, and other degenerative neurological diseases [1, 7]. Furthermore, it is imperative to evaluate the impact of dysphagia on patients’ quality of life and to use assessment tools in their native language to ensure greater acceptance [8]. Recent studies have highlighted the critical role of both Fiberoptic Endoscopic Evaluation of Swallowing (FEES) and Videofluoroscopy (VF) in the assessment of dysphagia, which are usually performed sequentially [7, 9, 10]. However, the simultaneous use of VF and FEES poses significant technical difficulties [11–13]. In addition, consistency between reviewers is often low and agreement between these diagnostic methods is limited, making accurate dysphagia assessment challenging [10]. Various studies have employed screening tests involving different fluid consistencies and food textures to assess and manage dysphagia [14, 15]. In the past, self-reported surveys such as the Dysphagia Handicap Index (DHI), Swallowing Quality of Life Questionnaire (SWAL-QOL), and M.D. Anderson’s Dysphagia Inventory (MDADI) have been used in cross-sectional studies and have garnered positive ratings [16]. Among them, the DHI is preferred because of its comprehensive structure and uniform evaluation in measuring the quality of life of dysphagia patients [9]. The DHI was introduced in 2012, and comprises 25 items segmented into three subscales: physical, emotional, and functional, addressing quality of life. Each item is responded to with “never,” “sometimes,” or “always,” with scores of 0, 2, and 4, corresponding to the respondents’ knowledge. The questionnaire concludes with a self-assessment on a 7-point scale, with 1 denoting normal, 4 signifying moderate, and 7 defining severe dysphagia [17]. Additionally, it serves as a tool for Patient-Reported Outcome Measures (PROMs) or Health-Related Quality of Life (HRQoL) [18]. Fundamentally, a few studies demonstrated the concept of quality of life which encompasses all aspects of an individual’s life, whereas Health-Related Quality of life (HRQoL) focuses on the aspects of QoL of an individual that are mostly impacted by ill health [18, 19]. However, the SWAL-QOL is widely regarded as the primary dysphagia assessment [20], with both the DHI and SWAL-QOL scoring the highest in psychometric evaluation and test interpretability across validity categories [7, 16]. However, the DHI is more advantageous from a clinical standpoint as it includes variables directly related to the patient’s symptoms [17]. Speech and Language Therapists (SLTs) play an important role in the clinical domain [21], consequently, effective screening and diagnostic methods need to be developed in the early stages to promote optimized intervention and goal-setting for dysphagia [22]. While there are few methods available for screening dysphagia, relying completely on tools from other countries cannot be considered enough for effectively identifying such patients, particularly due to the lack of a Bangla-specific form. A standardized and culturally acceptable diagnostic method is required for a thorough diagnosis of swallowing difficulties. As a result, this study aimed to culturally adapt and validate the Bangla version of DHI to address this need.

## Methods

A cross-sectional study design was chosen for this study.

### Translation

The DHI questionnaire was translated into Bangla to comply with the Cross-Cultural Adaptation of Self-Report Measures guidelines [23]. In the beginning, two experienced bilingual translators, Mrs. N. (MRS, BSLT) and Mrs. A. (BA, MA in English), who were native, separately translated the questionnaire. An impartial reviewer, Mr. M. (BA, MA in linguistics), merged their translations into a single version. This unified Bangla version was subsequently translated back into English by two other experienced bilingual translators, Mrs. S. (BPT, MPH) and Mrs. Sh. (BOT, MRS, Editor BJOTR). The back translation was compared with the original DHI by an expert in English Mrs. A (BA, MA in English). Finally, the back-translated version was sent to the investigators for review and feedback. An expert panel consisting of two (2) forward and two (2) backward translators, a linguist, and an English specialist was responsible for reviewing and selecting appropriate terminology to ensure cultural sensitivity. Content validity was determined using the Item-Objectives-Congruence (IOC). The Bangla version of the DHI was pilot-tested on five literate dysphagia patients of CRP who completed the questionnaire independently.

### Subjects

Between May 2019 and January 2020, subjects were recruited from the outpatient and inpatient units of the clinical Speech and Language Therapy department of Centre for the Rehabilitation of the Paralyzed (CRP), Bangladesh. Individuals aged 18 years or older [24], diagnosed with different types of neurological diseases accompanied by dysphagia, and who exhibit willingness to participate through consent, were selected through purposive sampling. Exclusion criteria included poor cognitive function and inadequate knowledge of Bangla. The study included 50 adult dysphagia patients, encompassing 34 males and 16 females. These patients had diverse neurological diagnoses such as stroke, head injury, cerebral palsy, and Parkinson’s disease, in which 41 were educated and 9 with no formal education. The control group consisted of 50 healthy persons (30 males and 20 females), who were recruited from caregivers, staff, and the general community and had no history of swallowing difficulties or neurological illnesses. The reliability of the DHI-Ban was assessed by readministering it to 18 dysphagia patients two weeks after their initial assessment without any swallowing treatments. Additionally, seven individuals were selected to serve as an expert panel for translations, IOC scores, and reviews.

### Dysphagia evaluation protocol 

The clinician assessed swallowing by directlyobserving the patient's ability to swallow variousconsistencies of food (Solid, minced, or pureed) andliquids (thick, or thickened). To precise this procedure,patients were encouraged to eat independently or withassistance if necessary. All patients were allowed toswallow without verbal instructions.

#### Validation and statistical testing

The validation of the DHI-Ban involved a thorough process, starting with evaluating content validity using IOC assessment to ensure compliance with linguistic and cultural standards (IOC ≥ 0.50). Internal consistency was assessed using Cronbach’s *α* coefficient, where α ≥ 0.7 is considered as a standard reliability coefficient. Test-retest reproducibility was measured by calculating the Intraclass Correlation Coefficient (ICC). Scores of ICC below 0.5 are indicative of poor reliability, while scores ranging from 0.5 to 0.75 suggest moderate reliability. Scores between 0.75 and 0.9 are deemed to reflect good reliability, and those exceeding 0.9 demonstrate excellent reliability [25]. A Wilcoxon two-sample test was used to compare continuous variables between the patient and healthy control groups across the three subscales and the total DHI-Ban score. Construct validity was evaluated by using the Spearman correlation coefficient to examine the relationship between the total DHI-Ban score and each item. All statistical tests were two-tailed, and *P* < 0.05 was considered statistically significant.

## Results

**Fig. 1:**
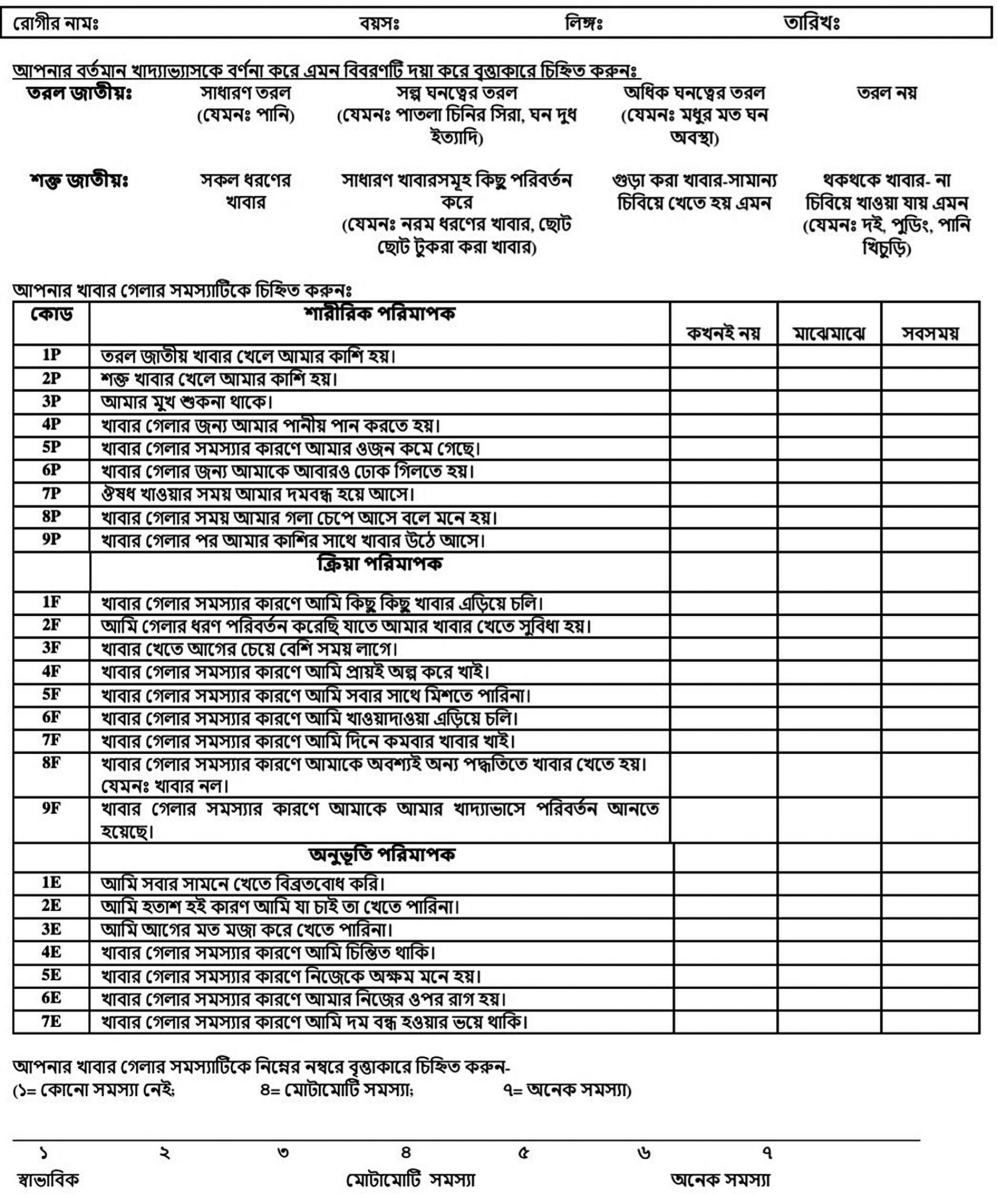
The Bangla version of Dysphagia Handicap Index (DHI-Ban)

The Bangla version of Dysphagia Handicap Index (DHI-Ban) is presented in Fig. 1.

The features of the total DHI-Ban score and subscale’s score are shown in Table 1. The study group consisted of 50 patients with dysphagia (34 males, 16 females) where mean age was 54 ± 13. The healthy people group included 50 healthy volunteers with a mean age 39.6 ± 12.6.

**Table 1:** Features of DHI-Ban subscale distribution of the patient group

DHI-Ban scale	No. of items	Possible range	Observed range	Mean	Median	SD
Total	25	0–100	10–98	49.3	49	21.1
Physical	9	0–36	2–36	15.4	14	8.1
Functional	9	0–36	2–36	19.3	21	9.3
Emotional	7	0–28	0–28	14.7	14	7.2

In this study, the frequency rate of male participants is higher than females. Approximately 68% were male and 32% were female in the patient group. And a large number of participants were strokes (86%). In addition, most of them were highly educated (22.4%) and approximately 54% were oral dysphagia (Table 2). Table 3 shows IOC scoring to check the content validity. All items got an accepted score of 0.67 to 1, indicating good content validity.

The internal consistency of the DHI-Ban for total score in this study was 0.89, indicating an excellent score and good for three subscales named physical, functional, and emotional presented scores of 0.74, 0.80, and 0.75.

**Table 2:** Distribution of demographic profile of patient group based on medical diagnosis, dysphagia evaluation criteria, gender and educational level

Medical diagnosis		Dysphagia evaluation criteria	
Stroke	86% (43)	Oral: 54% (27)	Showed anterior spillage, poor coordination and speed of chewing, and oral residue
Parkinson’s disease	8% (4)	Pharyngeal: 10% (5)	Showed delayed swallow, poor hyolaryngeal excursion, re-swallow, coughing and throat clearing, changes in breathing and voice
Head injury	4% (2)	Oro-pharyngeal: 36% (18)	Mixed of oral and pharyngeal dysphagia [26, 27]
Cerebral palsy	2% (1)		

Educational level		Gender	
No formal education	18.4%	Male (34)	Female (16)
Class 1–9	18.4%	27	16
SSC	20.4%	4	0
HSC	20.4%	2	0
Higher education	22.4%	1	0

**Table 3:** IOC scoring for content validity

	Serial	Item code	IOC Score	Accepted(+) Rejected(-)
Physical	1	1P	3/3 = 1	+
	2	2P	3/3 = 1	+
	3	3P	3/3 = 1	+
	4	4P	3/3 = 1	+
	5	5P	3/3 = 1	+
	6	6P	3/3 = 1	+
	7	7P	3/3 = 1	+
	8	8P	2/3 = 0.67	+
	9	9P	3/3 = 1	+
Functional	10	1 F	3/3 = 1	+
	11	2 F	3/3 = 1	+
	12	3 F	3/3 = 1	+
	13	4 F	3/3 = 1	+
	14	5 F	3/3 = 1	+
	15	6 F	3/3 = 1	+
	16	7 F	3/3 = 1	+
	17	8 F	2/3 = 0.67	+
	18	9 F	3/3 = 1	+
Emotional	18	1E	3/3 = 1	+
	20	2E	3/3 = 1	+
	21	3E	3/3 = 1	+
	22	4E	3/3 = 1	+
	23	5E	3/3 = 1	+
	24	6E	3/3 = 1	+
	25	7E	3/3 = 1	+

*P* Physical, *F* Functional, *E* Emotional, *IOC* Item-Objective-Congruence

**Accepted value- 1 ≥ 0.67; rejected value 0.33 ≥ 0

The test-retest reproducibility scored in this study was 0.84 which indicates an excellent score and also good for three subscales scored 0.86, 0.75 and 0.82 (Table 4).

**Table 4:** Comparison of the internal consistency and reproducibility between the DHI-Ban and the DHI

	DHI-Ban		DHI	
	Internal consistency (Cronbach’s α)	Test-retest reproducibility (ICC)	Internal consistency (Cronbach’s α)	Test-retest reproducibility (ICC)
Total	0.89	0.86	0.94	0.83
Physical	0.74	0.86	0.78	0.77
Functional	0.80	0.75	0.91	0.86
Emotional	0.75	0.82	0.86	0.75

*ICC* Intraclass correlation coefficient, *DHI* Dysphagia Handicap Index

DHI-Ban score was significantly higher for patients with dysphagia compared to the healthy people group (median 49, IQR 33.5–66 for the patient group compared to 1, IQR 0–6 for healthy people group; Z = 6.15, *p* < 0.001). Again comparison score of the three subscales, the score of physical (median 14, IQR 9.5–24 compared to 0 which lies between the range (0–4); Z = 5.913, *p* < 0.001), functional (IQR 21 that lies between the range from 11.5 to 26 compared to 0, IQR 0–0; Z = 6.16, *p* < 0.001) and as well as emotional (IQR was 14 lies between 10 and 20 compared to 0, IQR ranges 0–0; Z = 6.102; *p* < 001) (Table 5).

**Table 5:** Comparison of DHI-Ban score between dysphagia patient and healthy people group

	Dysphagia patients	Healthy people group
Total DHI-Ban	49 (33.5–66) 49.3 ± 21.1	1 (0–6) 3.04 ± 4.24
Physical	14 (9.5–24) 15.4 ± 8.1	0 (0–4) 2.2 ± 2.3
Functional	21 (11.5–26) 19.3 ± 9.3	0 (0–0) 0.3 ± 1.1
Emotional	14 (10–20) 14.7 ± 7.2	0 (0–0) 0.44 ± 1.1

**Values are given as Mean ± SD, *SD* Standard Deviation, *DHI* Dysphagia Handicap Index

Table 6, shows the spearman’s correlation coefficients of the subscales where in physical, there has found strong significant relationship where DHI-Ban score 0.809 where P value is 0.000 (<0.01). And in functional there was also significant relationship, where the DHI-Ban score was 0.924 and 0.000 (<0.01). In the emotional subscales score was 0.772 and significance was 0.000 (<0.01); which indicates the significant response from the participants. At the end of the DHI-Ban form is the self-reported severity.

**Table 6:** Spearman’s correlation between DHI-Ban’s 25 questions and total DHI-Ban in the patient group

	Spearman’s rho	DHI-Ban total
P1	Correlation coefficient	0.333
	Sig. (2-tailed)	0.018
P2	Correlation coefficient	0.451
	Sig. (2-tailed)	0.001
P3	Correlation coefficient	1.95
	Sig. (2-tailed)	1.75
P4	Correlation coefficient	0.369
	Sig. (2-tailed)	0.008
P5	Correlation coefficient	0.410
	Sig. (2-tailed)	0.003
P6	Correlation coefficient	0.593
	Sig. (2-tailed)	0.000
P7	Correlation coefficient	0.659
	Sig. (2-tailed)	0.000
P8	Correlation coefficient	0.602
	Sig. (2-tailed)	0.000
P9	Correlation coefficient	0.528
	Sig. (2-tailed)	0.000
F1	Correlation coefficient	0.607
	Sig. (2-tailed)	0.000
F2	Correlation coefficient	0.407
	Sig. (2-tailed)	0.003
F3	Correlation coefficient	0.599
	Sig. (2-tailed)	0.000
F4	Correlation coefficient	0.631
	Sig. (2-tailed)	0.000
F5	Correlation coefficient	0.392
	Sig. (2-tailed)	0.005
F6	Correlation coefficient	0.683
	Sig. (2-tailed)	0.000
F7	Correlation coefficient	0.705
	Sig. (2-tailed)	0.000
F8	Correlation coefficient	0.483
	Sig. (2-tailed)	0.000
F9	Correlation coefficient	0.642
	Sig. (2-tailed)	0.000
E1	Correlation coefficient	0.533
	Sig. (2-tailed)	0.000
E2	Correlation coefficient	0.481
	Sig. (2-tailed)	0.000
E3	Correlation coefficient	0.604
	Sig. (2-tailed)	0.000
E4	Correlation coefficient	0.463
	Sig. (2-tailed)	0.001
E5	Correlation coefficient	0.465
	Sig. (2-tailed)	0.001
E6	Correlation coefficient	0.547
	Sig. (2-tailed)	0.000
E7	Correlation coefficient	0.511
	Sig. (2-tailed)	0.000
Physical	Correlation coefficient	0.809
	Sig. (2-tailed)	0.000
Functional	Correlation coefficient	0.924
	Sig. (2-tailed)	0.000
Emotional	Correlation coefficient	0.772
	Sig. (2-tailed)	0.000

Correlation is significant at the <0.01 level; *P* Physical, *F* Functional, *E* Emotional

Table 7 presents the total score and the scores for each domain according to the self-perceived dysphagia severity. Table 8 represents different DHI translations and their comparison. A slightly higher score is found in the Bangla version of total DHI than in other translated DHI versions.

**Table 7:** DHI-Ban scores distribution according to self-perceived dysphagia severity of patient group

	Normal	Mild	Moderate	Severe
Total DHI	0.2 ± 0.6	3.0 ± 2.6	3.20 ± 3.24	5.44 ± 7.0
Physical	0.8 ± 0.3	1.20 ± 1.21	0.92 ± 1.9	1.24 ± 2.51
Functional	0.1 ± 0.24	0.74 ± 1.23	1.00 ± 1.8	2.7 ± 3.2
Emotional	0.1 ± 0.24	1.04 ± 1.23	1.16 ± 2.0	1.6 ± 2.9

Values are given as Mean ± SD, *SD*-Standard Deviation, *DHI*: Dysphagia Handicap Index

**Table 8:** Comparison among the scores of different DHI translation studies

	Bangla- DHI	DHI [17]	Hebrew- DHI [7]	Persian-DHI [5]	Arabic DHI [8]	Japanese DHI [9]
Total DHI	49 (33.5–66) 49.3 ± 21.1	- 27.33 ± 21.2	39 (18–56) 38.44 ± 24.4	4 (0–28) 6.53 ± 5.8	- 32.5 ± 24.7	10 (2–24) -
Physical	14 (9.5–24) 15.4 ± 8.1	- 11.52 ± 6.9	14 (10–20) 14.4 ± 8.2	11 (0–33) 15.23 ± 7.9	- 13.3 ± 9.6	4 (0–10) -
Functional	21 (11.5–26) 19.3 ± 9.3	- 10.04 ± 9.9	12 (4–20) 13.4 ± 9.9	12 (4–20) 13.4 ± 9.9	- 12.3 ± 10.1	2 (0–8) -
Emotional	14 (10–20) 14.7 ± 7.2	- 5.8 ± 6.8	8 (2–16) 10.1 ± 8.6	8 (2–16) 10.1 ± 8.6	- 6.9 ± 7.4	2 (0–8) -

**Values are given either as median and interquartile range or mean ± standard deviation or both; *DHI* Dysphagia Handicap Index

## Discussion

Developing effective assessment tools for assessing the quality of life of people who have major swallowing difficulties is of paramount importance. These tools play a crucial role in capturing the true impact of dysphagia on patients’ day-to-day activities and in shaping the development of focused interventions. Building upon traditional assessment methods, contemporary healthcare professionals incorporate diverse approaches to assess voice and swallowing problems. This holistic approach not only covers the clinical diagnosis but also emphasizes the subjective experiences of patients with dysphagia, thus influencing the development of tailored and efficient treatment plans. It is evident that the DHI is a reliable instrument for addressing the physical, functional, and emotional aspects of dysphagia, demonstrating excellent validity and reliability. The goal of this study was to linguistically validate and adapt the DHI for use in the Bangladeshi context. Initially, the forward translation process illustrated the need for explanatory words for question 8 in the physical subscale (strangling) and questions 5 and 7 in the emotional subscale (handicapped and choked). To ensure comprehension, the investigator conducted an IOC to test content validity. After expert evaluation and revisions, the approved validity score was ≥0.67, suggesting significant acceptability. Previously, a study focused on development and validation, researchers [28] found that expert panel members rated each item to calculate the IOC score and calculated content validity. Likewise, another research study [29], implemented IOC guidelines to check content validity. During the process of back translation, the terms ‘strangling’ and ‘handicapped’ were replaced with ‘feeling tight’ and ‘impaired/disabled,’ while retaining their original meaning. The expert panel performed an important discussion to finalize the preliminary version following these principles. The adaptation process concluded with the field test, in which the pre-final version was employed by following the guidelines of the previous study [23].

The research study comprised fifty individuals diagnosed with dysphagia. Of them, approximately 54% of the participants experienced oral dysphagia. The extensive nature of the assessment tool required participants to self-scoring, so educational background was a critical factor to consider. The questionnaire was completed by respondents with varying literacy levels, with assistance from caregivers provided for those who were illiterate. Earlier research has pointed out the important role of family members, caregivers, and others in assisting illiterate patients [7, 30]. Investigators conducted a Cronbach’s *α* test to assess internal consistency. It is noteworthy that, the functional domain had higher scores compared to the physical and emotional domains. In studies related to adaptation and translation, higher scores were noted in the physical domain compared to the functional and emotional domains [7, 8, 17, 31]. The DHI-Ban exhibited a Cronbach’s *α* score of 0.89, indicating good internal consistency comparable to the original DHI (*α* = 0.94) [17], as well as to the Arabic (*α* = 0.95) [8], Hebrew (*α* = 0.96) [7], Japanese (*α* = 0.95) [9], and Persian (*α* = 0.88) [5] versions. In addition, investigators used intra-class correlation (ICC) to evaluate the test-retest reproducibility. A 1-to 2-week interval was taken to analyze the correlation between pre-test and post-test scores, which aligned with previous research studies. Analysis of the ICC for the DHI-Ban questionnaire revealed a “good” level of reliability, ranging from 0.75 to 0.90, consistent with previous research [25]. Furthermore, both Cronbach’s *α* and ICC scores were compared between the DHI-Ban tool and the original DHI to assess their relative reliability and consistency. The results emphasize the significance of considering both the overall values and individual items within the DHI scale consistent with previous studies, significant responses were evident in all three domains (physical, functional, and emotional) of the DHI-Ban.

The DHI effectively distinguishes between individuals with dysphagia and those without dysphagia based on the clinical severity of dysphagia and demonstrates strong internal validity and sensitivity to significant changes in score [17]. Similarly, the Bangla version of the DHI (DHI-Ban) has exhibited comparable effectiveness in distinguishing the clinical severity of dysphagia, alongside maintaining strong psychometric properties through meaningful changes in scores. Participants rated their severity for 25 DHI-Ban questions and self-reported their symptoms without providing detailed information. The severity scale ranged from mild to severe, with some normal severity scores, similar to comparisons to the original and other translations. A comparison of the total DHI-Ban and its subscales revealed a significant association through Spearman’s correlation coefficients. Some items required multiple readings, possibly due to participants’ different educational backgrounds and cultural norms. Within the functional subscale, the phrase “I eat less due to swallowing problems” was incorrectly interpreted by participants as referring to food quantity rather than the intended meaning of eating frequency. The final version of DHI-Ban was tested on dysphagic patients and its effectiveness was confirmed; clinicians found that using synonyms and food examples improved understanding. Most participants found the DHI-Ban easy to understand, culturally appropriate, and beneficial. For the results, comparisons were made between different translation studies on DHI.

## Conclusion

The DHI-Ban is a comprehensive and effective dysphagia evaluation that has been culturally adapted to address the challenges that healthcare providers have when appropriately diagnosing dysphagia. Patients who speak Bangla as their primary language will benefit the most from this adaption, which allows healthcare professionals to deliver better care and make informed choices about patient treatment. Beyond that, the DHI-Ban is regarded as the most reliable self-assessment tool for determining how dysphagia impacts the quality of life in Bangladesh, offering an effortless and efficient solution.

### Limitations

The authors only included studies published shortly before this investigation. Besides, it is acknowledged that the exclusion of current studies coupled with limited study duration and resources represent substantial limitations of this study.

## Data Availability

I declare that the collected data are original and all the data are collected by authors. And the data cannot be shared due to maintain ethical consideration as well as participant confidentiality.

## Declarations

I the undersigned declare that the manuscript is original, has not been published anywhere before and is not currently being considered for publication elsewhere.
